# Brain White Matter Correlates of Creativity in Schizophrenia: A Diffusion Tensor Imaging Study

**DOI:** 10.3389/fnins.2020.00572

**Published:** 2020-06-23

**Authors:** Agurne Sampedro, Javier Peña, Naroa Ibarretxe-Bilbao, Alberto Cabrera-Zubizarreta, Pedro Sánchez, Ainara Gómez-Gastiasoro, Nagore Iriarte-Yoller, Cristóbal Pavón, Natalia Ojeda

**Affiliations:** ^1^Department of Methods and Experimental Psychology, Faculty of Psychology and Education, University of Deusto, Bilbao, Spain; ^2^OSATEK, MR Unit, Hospital of Galdakao, Galdakao, Spain; ^3^Refractory Psychosis Unit, Hospital Psiquiátrico de Álava, Vitoria-Gasteiz, Spain; ^4^Department of Neuroscience, Psychiatry Section, Faculty of Medicine and Odontology, University of the Basque Country (UPV/EHU), Leioa, Spain

**Keywords:** creativity, schizophrenia, white matter, divergent thinking, psychosis, fractional anisotropy

## Abstract

The relationship between creativity and psychopathology has been a controversial research topic for decades. Specifically, it has been shown that people with schizophrenia have an impairment in creative performance. However, little is known about the brain correlates underlying this impairment. Therefore, the aim of this study was to analyze whole brain white matter (WM) correlates of several creativity dimensions in people with schizophrenia. Fifty-five patients with schizophrenia underwent diffusion-weighted imaging on a 3T magnetic resonance imaging machine as well as a clinical and a creativity assessment, including verbal and figural creativity measures. Tract-based spatial statistic, implemented in FMRIB Software Library (FSL), was used to assess whole brain WM correlates with different creativity dimensions, controlling for sex, age, premorbid IQ, and medication. Mean fractional anisotropy (FA) in frontal, temporal, subcortical, brain stem, and interhemispheric regions correlated positively with figural originality. The most significant clusters included the right corticospinal tract (cerebral peduncle part) and the right body of the corpus callosum. Verbal creativity did not show any significant correlation. As a whole, these findings suggest that widespread WM integrity is involved in creative performance of patients with schizophrenia. Many of these areas have also been related to creativity in healthy people. In addition, some of these regions have shown to be particularly impaired in schizophrenia, suggesting that these WM alterations could be underlying the worse creative performance found in this pathology.

## Introduction

The relationship between creativity and schizophrenia has been a research topic of interest for centuries (Thys et al., 2013) since it was considered a key component for daily life problem solving (Plucker et al., 2015), and it seemed to have an impact on school, academic, and job performance (Rindermann and Neubauer, 2004). Although there are single famous cases of highly creative people with a possible diagnosis of schizophrenia (e.g., Vincent Van Gogh and John Nash), most patients do not show a higher level of creativity when compared to healthy people (Abraham et al., 2007). A recent meta-analysis from Acar et al. (2017) concludes that most evidence from empirical studies suggests that people with schizophrenia have, in fact, a worse creative performance, while very few studies have found a better creative performance in people with this disease (Glicksohn et al., 2001; Lauronen et al., 2004).

The study of the underlying neuroanatomical substrates of creativity in schizophrenia, nevertheless, is very scarce (Folley, 2006; Son et al., 2015), and it is only focused on specific brain areas and the fluency dimension instead of other aspects of creativity, such as originality. These studies (Folley, 2006; Son et al., 2015) did not find any significant results with creativity. In contrast, the amount of studies that have analyzed the underlying neuroanatomical substrates of creativity among healthy people has been growing during the last decade (Kenett et al., 2018). However, the brain correlates of creativity still remain inconclusive (Arden et al., 2010; Sawyer, 2011; Takeuchi and Kawashima, 2018). Some of the main brain regions that have been related to human creative thinking among healthy people include prefrontal, parietal, temporal, and subcortical areas (Arden et al., 2010; Boccia et al., 2015; Wu et al., 2015; Abraham et al., 2018; Japardi et al., 2018; Shi et al., 2018; Vartanian et al., 2018; Sun et al., 2019). Most of these findings have come from functional magnetic resonance imaging (fMRI) studies in healthy people.

Regarding structural brain correlates of creativity in healthy people, contradictory and inconclusive findings have been found (Takeuchi and Kawashima, 2018). Specifically, very few studies have analyzed the association between WM structural connectivity and creativity. Studies examining FA have found both positive (Takeuchi et al., 2010b) and negative associations (Jung et al., 2010), as well as non-significant associations (Takeuchi et al., 2016). For instance, Jung et al. (2010) found a negative correlation between a composite creativity score (including verbal and figural creativity tasks) and FA mainly within the left inferior frontal WM. In contrast, Takeuchi et al. (2010b) found positive correlations between total creativity (obtained from three verbal creativity tasks) and FA from the bilateral prefrontal cortex, corpus callosum, cingulate cortex, bilateral basal ganglia, bilateral temporoparietal junction, and the right inferior parietal lobe. Findings from Takeuchi et al. (2010b) suggest that multiple brain regions are involved in general creative thinking. Moreover, these results support the idea that both intra- and interhemispheric connections (especially the corpus callosum) as well as the frontal lobe underlie creative thinking. The role of intra- and interhemispheric connectivity is supported by the long-standing idea that integration of information and specifically integration of distant ideas are important for creativity (Katz, 1986; Hoppe and Kyle, 1990; Takeuchi et al., 2010b; Razumnikova and Volf, 2015). Concerning the frontal lobe, it has been proposed that WM integrity in this region facilitates multiple high-level cognitive functions, such as working memory and executive functions, which seem to underlie creative thinking (Takeuchi et al., 2010b). Additional evidence of the role of frontal lobe comes from studies that investigated the effect of transcranial direct current stimulation on creativity (Lucchiari et al., 2018).

Interestingly, brain regions identified by studies analyzing structural brain correlates of creativity in healthy individuals (Jung et al., 2010; Takeuchi et al., 2010b) are regions that have shown to be impaired in schizophrenia (Ellison-Wright and Bullmore, 2009; Bora et al., 2011; Stämpfli et al., 2019). This could suggest that creativity alterations found in this pathology could be at least partially due to WM abnormalities. In fact, structural connectivity alterations are a core characteristic of this disease (Stämpfli et al., 2019). Thus, WM alterations in schizophrenia are mainly circumscribed to frontal and temporal regions including mainly interhemispheric (corpus callosum), intrahemispheric (e.g., thalamic radiation, superior longitudinal fasciculus, inferior longitudinal fasciculus, inferior fronto-occipital fasciculus, and fornix), and projective fibers (corticospinal tract) (Ellison-Wright and Bullmore, 2009; Bora et al., 2011; Wheeler and Voineskos, 2014; Stämpfli et al., 2019).

Taking into account the structural connectivity alterations as well as the creativity impairment shown in schizophrenia, it seems relevant to study the WM correlates of creativity in this disease. As far as the authors are aware, to date, none of the previous studies have investigated whole brain WM correlates with different dimensions of creativity (such as fluency and originality) in schizophrenia. Therefore, the objective of this exploratory study was to assess whole brain WM correlates of several creativity dimensions in people with schizophrenia. Based on previous results from Takeuchi et al. (2010b), our hypothesis was that creativity would be positively associated mainly with the frontal lobe as well as with interhemispheric WM fibers.

## Materials and Methods

### Participants

The sample consisted of 55 patients [47 men and eight women, mean age 41.22 (*SD* = 10.41)] diagnosed with schizophrenia and recruited from the Psychiatric Hospital of Álava and the Community Mental Health Services in Álava (Basque Country, Spain). All patients met the diagnostic criteria for schizophrenia according to the Structured Clinical Interview for Diagnostic and Statistical Manual of Mental Disorders, Fourth Edition, Text Revision (DSM-IV-TR; American Psychiatric Association, 2000).

Exclusion criteria consisted of: (a) clinical instability (total score in PANSS-Positive >19), (b) cognitive impairment secondary to another disease, (c) main diagnosis of a substance use disorder or presenting active drug consumption at the time of the study, (d) relevant modifications to the antipsychotic drug treatment in the previous 3 months, (e) diagnosis of an active major affective disorder, and (f) incompatibilities with magnetic resonance imaging: claustrophobia, metal implants in the body, or patients who were undergoing deep brain stimulation. The study protocol had the approval of the Clinical Research Ethics Committee of the Autonomous Region of the Basque Country (CEIC-E) in Spain (PI2017044). The trial was registered in clinicaltrials.gov (NCT03509597). All participants took part in the study voluntarily and provided their written informed consent. Participants did not receive any monetary reward for taking part in the study.

### Measures

#### Creativity

Creativity was measured by means of two subtests from the Torrance Test of Creative Thinking (Torrance, 1966). From the Verbal Form of the test, the Unusual Uses subtest was administered. In this test, participants were asked to write all the unusual uses for cardboard boxes that they could think of. Three dimensions were measured: (1) originality, (2) fluency, and (3) flexibility. The Picture Completion subtest was used from the Figural Form of the test. In this activity, participants were asked to complete 10 unfinished figures, generating as many ideas as possible. Six dimensions were measured: (1) originality, (2) elaboration, (3) fluency, (4) resistance to premature closure, (5) abstractness of titles, and (6) figural creative strengths. Additionally, the flexibility dimension was measured using the criteria from the Spanish adaptation of the Torrance Test of Creative Thinking (Jiménez et al., 2007). Participants were given 4 min to complete each creative task. An expert neuropsychologist corrected all the tests.

#### Clinical Symptoms

Psychopathology was assessed with the PANSS (Kay et al., 1987). Positive Scale, Negative Scale, and General Psychopathology Scales were provided.

#### Premorbid IQ

Premorbid IQ was calculated with the Accentuation Reading Test (TAP) (Del Ser et al., 1997), a Spanish version of the National Adult Reading Test (Nelson and Willison, 1991). For the estimation of premorbid IQ, raw scores were converted using the full scale IQ of Gomar et al. (2011).

#### Handedness

Handedness was measured using the Edinburgh Handedness Inventory (Oldfield, 1971). In this self-reported questionnaire, participants were asked to indicate their preference of hand use for 10 everyday activities. Responses for each activity could be no preference (0 points), preference (1 point), and very strong preference (2 points). Handedness consistency was calculated using a formula (right − left/right + left), and the obtained scores ranged from 100 (perfectly right-handed) to −100 (perfectly left-handed). Mixed-handed were considered those participants who obtained scores ranging from −79 to 79, and consistent-handed were those with scores ranging from −100 to −80 or from 80 to 100.

### Image Acquisition and Analysis

Diffusion-weighted images (DWIs) were obtained on a Philips 3T Achieva Dstream, in an axial orientation in an anterior–posterior phase direction using a single-shot EPI sequence (TR = 7,540 ms and TE = 76 ms, matrix size = 120 mm × 117 mm; flip angle = 90°, FOV = 240 × 240 × 130, slice thickness = 2 mm, no gap, 65 slices, acquisition time = 9′31′′, voxel size = 1.67 × 1.67 × 2.0) with diffusion weighting in 32 uniformly distributed directions (b = 1,000 s/mm^2^) and 1 b = 0 s/mm^2^.

The FMRIB Software Library (FSL) (Smith et al., 2004) version 5.0.11^1^ was used for the preprocessing and analysis of diffusion data. First, each participant’s images were concatenated and radiologically oriented. Next, data were corrected for head motion and eddy currents, brain extraction was performed using BET (Brain Extraction Tool) (Smith, 2002), and the diffusion gradients (bvecs) were rotated to be corrected accordingly. Then, FA, mean diffusivity (MD), radial diffusivity (RD), axial diffusivity (AD), and mode of anisotropy (MO) images were obtained by fitting a tensor model to the raw diffusion data using FDT (DTIFIT). Afterward, voxelwise statistical analysis of the data was carried out using Tract-Based Spatial Statistics (TBSS; Smith et al., 2006). FNIRT tool (Andersson et al., 2007a, b) was used to align all subjects’ FA data into a common space by combining the non-linear transform to the target FA image with the affine transform from that target to MNI152 space. Later, the mean FA image was created using a threshold of 0.2 and thinned to create a mean FA skeleton, which represented the centers of all tracts common to the group. Each participant’s aligned FA data were then projected onto this skeleton and the resulting data were fed into voxelwise cross-subject statistics. The “tbss non FA” script from TBSS was used to analyze MD, RD, MO, and, AD data. This applies the original non-lineal registration to the MD, RD, MO, or AD data, merges all subjects warped MD, RD, MO, or AD data into a 4D file, then projects this onto the original mean FA skeleton, and creates the 4D projected data.

### Data Analyses

All variables were tested for normality. Means and standard deviations were obtained using IBM SPSS version 26.0 (SPSS Inc., Chicago, IL, United States). As creativity variables did not follow a normal distribution, these were log-transformed (LN) and all variables were then transformed into Z scores. To examine the relationship between WM indexes and creativity, permutation-based inferences (5,000 permutations) with the threshold-free cluster enhancement (TFCE) correction method for multiple comparisons, including the – – T2 option, were carried out using FSL’s Randomise Tool (Winkler et al., 2014). Based on findings from previous literature (Bartzokis et al., 2003; Schmithorst et al., 2005; Szeszko et al., 2008; White et al., 2011; Ryman et al., 2014; Samartzis et al., 2014; Kubota et al., 2015; Takeuchi et al., 2016; Jensen et al., 2019), sex, age, premorbid IQ, and medication were entered as covariates in the regression analysis. The statistical threshold was set at *p* < 0.05 corrected for family wise error (FWE), with an extent threshold of 100 voxels. Effect sizes for each cluster of the correlations were calculated according to Cohen’s *d* formula. Cohen’s *d* of 0.20, 0.50, and 0.80 were considered small, medium, and large, respectively (Cohen, 1988). The maximum coordinates encompassed in the clusters and additional significant regions were visually inspected and located and later labeled anatomically with the MRI Atlas of Human White Matter (Oishi et al., 2010) as well as the JHU-ICBM-DTI-81 WM Labels and JHU White-Matter Tractography Atlas implemented in FSL.

Participants from the present study were assured that raw data would remain confidential and would not be shared. Therefore, data of the study are not available in the public domain.

## Results

### Sociodemographic, Clinical, and Creativity Data

Sociodemographic and clinical characteristics of the sample as well as performance in verbal and figural creativity are shown in Table 1. Medication was transformed to chlorpromazine through the defined daily dose method (Leucht et al., 2016; Rothe et al., 2018). Standardized mean creativity scores of patients with schizophrenia based on a sample of healthy adults (obtained from Sampedro et al., 2019) were the following: −0.55 (*SD* = 0.78) for verbal originality, −0.78 (*SD* = 0.61) for verbal fluency, −0.93 (*SD* = 0.69) for verbal flexibility, −0.19 (*SD* = 0.64) for figural originality, −0.20 (*SD* = 1.05) for figural fluency, −0.55 (*SD* = 0.81) for figural elaboration, −0.27 (*SD* = 0.99) for figural flexibility, −0.41 (*SD* = 1.02) for figural resistance to closure, −0.78 (*SD* = 0.74) for figural abstractness of titles, and −0.84 (*SD* = 0.57) for figural creative strengths.

**TABLE 1:** Sociodemographic, clinical, and creativity data of the sample.

	Patients with schizophrenia (*N* = 55) *M (SD)*
**Sociodemographic and clinical characteristics**	
Sex (male)	47(85.45%)
Age (years)	41.22 (10.41)
Education years	10.31(2.55
Handedness	
Right-handedness	41(74.55%)
Mixed	11(20%)
Age of onset	21.87 (6.28)
Previous hospitalizations	5.95 (6.19)
Premorbid IQ	96.40 (9.22)
Medication dosage (chlorpromazine equivalent doses, mg/day)	485.88 (295.47)
PANSS Positive	15.45 (5.76)
PANSS Negative	20.95 (7.14)
PANSS General	35.11 (9.77)
PANSS Total	71.50 (18.61)
**Creativity assessment**	
Verbal originality	5.19 (3.94)
Verbal fluency	8.85 (3.67)
Verbal flexibility	5.39 (2.4)
Figural originality	2.79 (1.67)
Figural fluency	6.50 (2.29)
Figural elaboration	19.50 (10.82)
Figural flexibility	5.32 (1.81)
Figural resistance to Closure	10.08 (4.00)
Figural abstractness of Titles	5.25 (4.54)
Figural creative strengths	2.85 (2.37)

**M*, mean; *SD*, standard deviation; PANSS, Positive and Negative Syndrome Scale.*

### Correlations Between Creativity and White Matter

**FIGURE 1:**
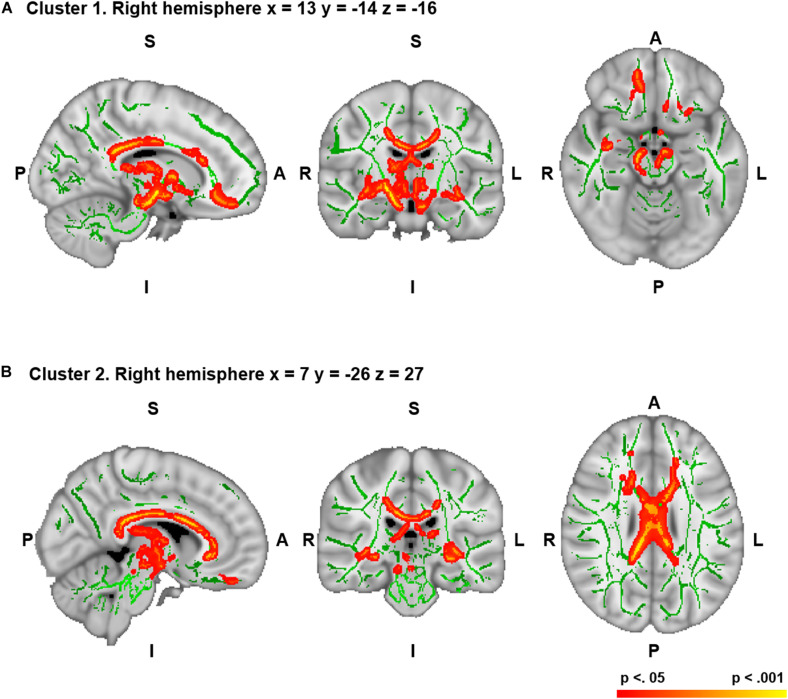
Correlations between figural originality and white matter (WM) mean fractional anisotropy (FA) in the two significant clusters [**(A)** for cluster 1 and **(B)** for cluster 2]. Correlations are significant at *p* < 0.05 corrected for family wise error (FWE). Significant WM regions are shown in red-yellow; the WM skeleton is shown in green. Significant voxels are thickened for easier visualization. S, superior; I, inferior; A, anterior; P, posterior; L, left; R, right. Coordinates are shown in Montreal Neurological Institute (MNI) space. The region located in the coordinates that showed the maximum significance was the right cerebral peduncle of the corticospinal tract for cluster 1 **(A)** and the right body of the corpus callosum for cluster 2 **(B)**.

Significant positive correlations were found between mean WM FA and figural originality in two clusters (Table 2 and Figure 1). Significant negative correlations were not found between creativity dimensions and any brain region. In addition, there were no significant correlations between mean FA and verbal creativity dimensions.

**TABLE 2:** Correlation analysis between figural originality and mean fractional anisotropy (FA) showing two significant clusters.

			FSL coordinates					
	Brain regions	Cluster size (voxels)	x	y	z	*t*	*p*	Effect size (Cohen’s *d*)
Cluster 1	Corticospinal tract (cerebral peduncle part) (R)	7,551	77	112	56	3.91	0.024	1.13
Cluster 2	Body of the corpus callosum (R)	5,681	83	100	99	3.25	0.025	0.92

*Cluster size denotes the extent of the cluster of significant voxels. FMRIB Software Library (FSL) coordinates indicate: x increases from left to right; y increases from posterior to anterior; and z increases from inferior to superior. Regions represent the maximum significant correlation coordinate encompassed in the given cluster. R, right.*

The subdomain of figural creativity that correlated positively with mean WM FA was originality. More specifically, WM fibers corresponding to the peak level of significance were located in the right corticospinal tract (cerebral peduncle part) for cluster 1 and the right body of the corpus callosum for cluster 2 (Table 2). Other WM areas comprised in those same significant clusters included bilateral frontal (uncinate fasciculus, inferior fronto-occipital fasciculus, and WM adjacent to the superior frontal gyrus and orbitofrontal cortex), right temporal (uncinate fasciculus and WM adjacent to the inferior temporal gyrus), bilateral subcortical (fornix, anterior thalamic radiation, lenticular fasciculus, internal capsule, and external capsule), left brain stem (corticospinal tract), and bilateral interhemispheric (body, genu, and splenium of the corpus callosum) regions (FWE-corrected, *p* < 0.05) (Figure 1). Effect sizes for these correlations were large for both clusters (Cohen’s *d* = 1.13 and Cohen’s *d* = 0.92). No significant correlations were found for MD, AD, RD, or MO in this variable.

## Discussion

The main objective of this study was to assess whole brain WM correlates of different creativity dimensions in people with schizophrenia. Our findings showed that creative performance was positively related to WM mean FA adjacent to multiple brain regions, including frontal, temporal, subcortical, and brain stem areas as well as to interhemispheric WM fibers. Significant positive correlations were specially found in figural originality but not in any verbal creativity dimension. As a whole, these results are quite consistent with findings from Takeuchi et al. (2010b), in which total creativity correlated positively with WM adjacent to prefrontal areas, temporal and parietal lobes, basal ganglia, and corpus callosum in healthy people.

As expected, significant positive correlations were found between the figural originality subdomain of creativity and FA in the corpus callosum. This is congruent with findings from previous studies analyzing WM correlates of creativity (Takeuchi et al., 2010b; Zeng et al., 2017). WM integrity in the corpus callosum is thought to promote interhemispheric information processing and communication (Schulte et al., 2005). In addition, according to Heilman et al. (2003), different forms of knowledge and cognitive strategies can be combined through interhemispheric communication and, consequently, facilitate creative thinking. Therefore, these results support the long-held idea that interhemispheric communication is relevant for creative thinking (Katz, 1986; Bogen and Bogen, 1988; Hoppe and Kyle, 1990; Lindell, 2011).

Consistent with our hypothesis, results also showed significant positive correlations between the figural originality subdomain of creativity and WM tracts traversing the frontal lobe, such as the uncinate fasciculus and WM adjacent to the prefrontal cortex. These findings were also in line with both structural WM studies (Takeuchi et al., 2010b) and fMRI studies (Beaty et al., 2014, 2018; Japardi et al., 2018; Marron et al., 2018; Sun et al., 2019). Studies analyzing the effect of transcranial direct current stimulation on creativity also support the frontal lobe’s role in creative performance (Lucchiari et al., 2018). Moreover, creativity has been particularly related to the prefrontal cortex (Benedek et al., 2014a; Beaty et al., 2018; Lucchiari et al., 2018). It has been suggested that several higher-order cognitive functions, such as working memory and executive functions, are required for creative thinking since they enable, for instance, the maintenance of novel information in an elevated state of activity (de Dreu et al., 2012) or the inhibition of salient but unoriginal ideas (Benedek et al., 2014b). WM integrity in the frontal lobe seems to be involved in these higher-order cognitive abilities (Charlton et al., 2010; Jacobs et al., 2013) and, therefore, in creative thinking (Takeuchi et al., 2010b). Figural originality was additionally related to WM adjacent to the orbitofrontal cortex, including the straight and orbital gyrus. The orbitofrontal cortex has been related to decision-making (Padoa-Schioppa and Conen, 2017), to attentional control (Ohtani et al., 2017), and to creativity in men (Abraham et al., 2014). According to Schuck et al. (2016), the orbitofrontal cortex is a region involved in the flexible up-to-date representation of information that is relevant to a given task, thus allowing decision-making. The association of this region with originality makes sense, since representing all the relevant information for a creative task seems to be important in order to find or decide which is the most original idea. Furthermore, this region seems to be particularly altered in patients with schizophrenia (Takayanagi et al., 2010; Kanahara et al., 2013).

In addition to WM from the corpus callosum and frontal lobe, we also found a relationship between the figural originality subdomain of creativity and intrahemispheric WM fibers located in several association regions from the temporal lobe, specifically, the uncinate fasciculus and WM adjacent to the inferior temporal gyrus. This indicates that intrahemispheric communication may also be required for creative thinking through the integration of remote information belonging to multiple regions that are involved in different domains (Takeuchi et al., 2010b). Besides, the involvement of the temporal lobe in creative thinking is consistent with other structural (Takeuchi et al., 2010b) and functional studies (Benedek et al., 2014a; Beaty et al., 2015) and could be due to its relation with memory retrieval. Memory retrieval is required for creative thinking, since original ideas seems to be generated through the direction of attention to internal knowledge representations, the controlled retrieval, and the recombination of this stored knowledge (Benedek et al., 2014a).

White matter tracts located in subcortical regions were also associated with creativity. They included the fornix, internal capsule, external capsule, anterior thalamic radiation, and lenticular fasciculus. These regions could be involved in creativity due to their implication in the dopaminergic system (Takeuchi et al., 2010a, b; Jauk et al., 2015). An increased WM integrity in these subcortical regions is thought to increase functional connectivity and, consequently, improve some higher-order frontal lobe abilities that underlie creative thinking, such as cognitive flexibility, through the regulation of the dopaminergic system (Takeuchi et al., 2010b).

Finally, a positive correlation was found between creativity and the corticospinal tract in its cerebral peduncle part. Although this region has not been related to creativity, the cerebral peduncle is a main component of the cortico-ponto-cerebello-thalamo-cortical loop and it is believed to connect the cerebellum with the neocortex (Hüttlova et al., 2014). The cerebral peduncle and the corticospinal tract are regions involved not only in movement but also in cognitive processes such as coordination and information processing (Picard et al., 2008). Additional evidence comes from several studies that have shown that the cerebellum is highly involved in creativity through cerebral–cerebellar interactions that underlie implicit manipulation of mental representation, which in turn promote the development of more creative ideas (Saggar et al., 2015, 2017). Moreover, cerebral peduncles and the corticospinal tract are particularly impaired in schizophrenia (Hüttlova et al., 2014; Stämpfli et al., 2019). Therefore, decreased WM integrity in these areas could possibly promote an impairment in some cognitive functions such as creativity.

Although we found some greater correlations in regions from the right hemisphere, in general, there were bilateral correlations with creativity. In fact, in spite of the long-held belief that the right hemisphere is mainly responsible for creative thinking (Harnad, 1972), nowadays, results from most brain imaging studies suggest that creativity requires both hemispheres (Beaty et al., 2014, 2016; Wu et al., 2015). Moreover, it seems that it is not so much a matter of the left or right hemisphere, but rather a complex set of intra- and interhemispheric connections within the whole brain (Corballis, 2018).

It is worth mentioning that we did not find significant correlations with all dimensions and, specifically, with verbal creativity. The absence of significant correlations in various variables could be partially due to the sample size. In any case, several of the few studies that analyze WM FA correlates of creativity have not found significant correlations either in patients with schizophrenia (Folley, 2006; Son et al., 2015) or in healthy people (Takeuchi et al., 2016). Other studies analyzing WM integrity (Zeng et al., 2017) or gray matter volume (Jauk et al., 2015) also found significant correlations with originality but not with fluency. An additional possible explanation for these results could be the language impairment present in schizophrenia (Mitchell and Crow, 2005; Docherty et al., 2011), which makes verbal creativity tasks particularly challenging for these patients. In consequence, the general performance in verbal dimensions may have been low, with a lower variability of the data among the sample than in figural creativity, making it more difficult to correlate with WM integrity. More research is required to analyze brain correlates of verbal creativity in schizophrenia, controlling for the possible influence of other cognitive variables, such as verbal memory.

Taken together, this study provides initial data for brain WM correlates of creativity in schizophrenia. Overall, results suggest that both intrahemispheric communication and interhemispheric communication seem to be required for creative thinking, allowing the integration of remote associations of ideas. Our findings support the role of the frontal lobe in creative thinking but also indicate the relevance of other brain regions such as the corticospinal tract. Results from this study are similar to those from Takeuchi et al. (2010b) carried out with healthy people, which suggests that similar brain regions are involved in creative thinking of people with schizophrenia and healthy people. This idea is in line with the creative cognition approach, which considers that creativity emerges from the application of basic cognitive functions to already existing knowledge structures and therefore assumes that creative capacity is a normative human characteristic (Ward, 2007). Given that WM alteration is a core characteristic of schizophrenia (Stämpfli et al., 2019) and that many of the brain regions that correlated with creativity in our study are brain regions that are particularly impaired in this disease (e.g., the corpus callosum, fornix, or corticospinal tract), we could hypothesize that the impairment in creative performance of people with schizophrenia is related to an alteration in WM integrity. Nevertheless, this idea must be considered with caution, since we did not compare WM integrity of our sample with healthy controls.

### Limitations

The present study has several limitations that should be considered. First, we did not include healthy controls to check whether there was an impairment in WM integrity. However, our interpretations of the results were based on previous evidence, which has shown that people with schizophrenia have WM alterations in specific brain regions (Ellison-Wright and Bullmore, 2009; Bora et al., 2011; Stämpfli et al., 2019). Future studies should explore WM correlates of creativity in schizophrenia compared to healthy controls to analyze whether there are differences in the brain areas related to creativity. Second, the small sample size limits generalization of results, so more studies should be conducted with larger samples and results of this study should be considered with caution. Finally, other variables such as clinical symptoms and cognitive capacity in several domains like executive functions, attention, or working memory could have an effect on creative performance; hence, future studies should control the influence of these variables.

### Conclusion

As far as the authors are aware, this is the first study analyzing whole brain WM correlates of different creativity dimensions in schizophrenia. We consider that this study provides relevant preliminary data that could, on the one hand, shed light on the long-standing and controversial association between psychosis and creativity and, on the other hand, contribute to fill a gap in research on the neuroscience of human creativity. Results suggest that multiple brain regions are involved in creative thinking, which makes sense due to the complexity and multifaceted nature of this higher-order cognitive function. Yet more research is needed in order to understand the neural bases of creativity in both schizophrenia and healthy people. Understanding the brain correlates of creativity in schizophrenia has relevant implications for the treatment of mental disorders and for the enhancement of this ability that is essential for the survival and enrichment of humankind (Carson, 2014).

## Data Availability Statement

The datasets for this article are not publicly available because participants from the present study were assured raw data would remain confidential and would not be shared.

## Ethics Statement

The studies involving human participants were reviewed and approved by Clinical Research Ethics Committee of the Autonomous Region of the Basque Country (CEIC-E) in Spain (PI2017044). The patients/participants provided their written informed consent to participate in this study.

## Author Contributions

NO, NI-B, JP, and PS designed the study and wrote the protocol. AS, AC-Z, PS, NI-Y, and CP performed the clinical, neuropsychological, and neuroimaging assessments. AS and JP managed the literature searches. AS, JP, and AG-G undertook the statistical analysis. AS and JP wrote the first draft of the manuscript. All authors contributed to the writing and revision of the manuscript and approved the final manuscript.

## Conflict of Interest

The authors declare that the research was conducted in the absence of any commercial or financial relationships that could be construed as a potential conflict of interest.

## Footnotes

1 https://fsl.fmrib.ox.ac.uk/fsl/fslwiki/FSL

## Abbreviations

FA: fractional anisotropy
PANSS: Positive and Negative Syndrome Scale
WM: white matter.
